# Blood Erythrocyte Concentrations of Cadmium and Lead and the Risk of B-Cell Non-Hodgkin’s Lymphoma and Multiple Myeloma: A Nested Case-Control Study

**DOI:** 10.1371/journal.pone.0081892

**Published:** 2013-11-28

**Authors:** Rachel S. Kelly, Thomas Lundh, Miquel Porta, Ingvar A. Bergdahl, Domenico Palli, Ann-Sofie Johansson, Maria Botsivali, Paolo Vineis, Roel Vermeulen, Soterios A. Kyrtopoulos, Marc Chadeau-Hyam, on behalf of the EnviroGenoMarkers project consortium

**Affiliations:** 1 Medical Research Council-Health Protection Agency Centre for Environment and Health, Department of Epidemiology and Biostatistics, Imperial College London, London, United Kingdom; 2 Department of Occupational and Environmental Medicine, Lund University Hospital, Lund, Sweden; 3 Hospital del Mar Research Institute - IMIM, CIBER Epidemiología y Salud Pública (CIBERESP) and Universitat Autònoma de Barcelona, Barcelona, Catalonia, Spain; 4 Occupational and Environmental Medicine, Department of Public Health and Clinical Medicine, Umeå University, Umeå, Sweden; 5 Molecular and Nutritional Epidemiology Unit, Cancer Prevention and Research Institute (ISPO), Florence, Italy; 6 Department of Radiation Sciences, Oncology, Umeå University, Umeå, Sweden; 7 National Hellenic Research Foundation, Institute of Biology, Pharmaceutical Chemistry and Biotechnology, Athens, Greece; 8 HuGeF Foundation, Turin, Italy; 9 Institute for Risk Assessment Sciences, Division of Environmental Epidemiology, Utrecht University, Utrecht, The Netherlands; University of Campinas, Brazil

## Abstract

**Background:**

Cadmium (Cd) and lead (Pb) are hypothesised to be risk factors for non-Hodgkin’s lymphoma (NHL), a group of haematological malignancies with a suspected environmental aetiology. Within the EnviroGenoMarkers study we utilised pre-diagnostic erythrocyte concentrations of Cd and Pb to determine whether exposure was associated with risk of B-cell NHL and multiple myeloma.

**Methods:**

194 incident cases of B-cell NHL and 76 cases of multiple myeloma diagnosed between 1990 and 2006 were identified from two existing cohorts; EPIC-Italy and the Northern Sweden Health and Disease Study. Cases were matched to healthy controls by centre, age, gender and date of blood collection. Cd and Pb were measured in blood samples provided at recruitment using inductively coupled plasma-mass spectrometry. Logistic regression was applied to assess the association with risk. Analyses were stratified by cohort and gender and by subtype where possible.

**Results:**

There was little evidence of an increased risk of B-cell NHL or multiple myeloma with exposure to Cd (B-cell NHL: OR 1.09 95%CI 0.61, 1.93, MM: OR 1.16 95% CI: 0.40, 3.40 ) or Pb (B-cell NHL: 0.93 95% CI 0.43, 2.02, multiple myeloma: OR 1.63 95%CI 0.45, 5.94) in the total population when comparing the highest to the lowest quartile of exposure. However, gender and cohort specific differences in results were observed. In females the risk of B-cell NHL was more than doubled in those with a body burden of Cd >1µg/L (OR 2.20 95%CI; 1.04, 4.65).

**Conclusions:**

This nested case-control study does not support a consistent positive association between Cd or Pb and NHL, but there is some indication of a gender specific effect suggesting further research is warranted.

## Introduction

Non-essential metals such as cadmium (Cd) and lead (Pb) are ubiquitous persistent environmental toxicants known to bioaccumulate in the human body. Both metals are highly toxic even in trace amounts [1], and the global burden of Cd and Pb remains high due to their widespread usage in industrial and manufacturing processes and through contaminated phosphate fertilisers [2–4]. Exposure occurs primarily through the ingestion of contaminated food and drinking water, the inhalation of contaminated air, and smoking [1,5,6]. Consequently, Pb and Cd constitute some of the most widespread environmental pollutants in the world and, with no known beneficial physiological role [1,7] they represent an important public health burden [5,6].

Since 1993, Cd and its compounds have been classified as group 1 carcinogens by the International Agency for Research on Cancer (IARC), based on sufficient evidence from animal models and increased incidence of breast and lung cancer in humans [8]. Pb is classified as a possible human carcinogen (group2b), while its inorganic compounds are deemed probable carcinogens (group 2a) [9] based on limited epidemiological evidence for an increased risk of lung, stomach, kidney and brain cancers [2]. Multiple molecular and cellular mechanisms are thought to contribute both to the carcinogenicity of Cd and to the suspected carcinogenicity of Pb in humans [10,11]. 

Non-Hodgkin’s lymphomas (NHL) are a heterogeneous group of lymphoid malignancies whose aetiology is largely unknown. The best characterised risk factor to date is severe immunodeficiency [12]. However, this accounts for only a small percentage of cases and evidence from epidemiological and occupational studies suggests an environmental aetiology. In particular, polychlorinated biphenyls (PCBs) and dioxins have been implicated in studies of occupationally exposed cohorts or populations living in the vicinity of industrial sources of these pollutants. This association is supported by the temporal relationship between worldwide usage and incidence trends of NHL [13]. Similarly, there is evidence for an increase of NHL incidence in populations living near refineries that emit Pb and Cd [14]. Both agents have been observed to modulate the immune system [3,15,16] and furthermore, a primary target of Pb [17] and a secondary target of Cd is the hematopoietic system [17,18], making these metals plausible candidate risk factors for NHL. 

The EnviroGenoMarkers Study is a case-control study nested within two prospective cohorts aiming at the development of a new generation of biomarkers to better characterise the relationship between exposure to environmental pollutants and adverse health effects. The aim of the present study was to analyse the relationship between pre-diagnostic blood erythrocyte concentrations of Pb and Cd and risk of B-cell NHL and multiple myeloma. 

## Materials and Methods

### Ethics Statement

This study was approved by the committees on research ethics in Umea (Dnr 08-215M) and in Florence (ref 347/2009) in accordance with the Declaration of Helsinki of the World Medical Association. All participants provided written consent at recruitment. 

### Study subjects

The EnviroGenoMarkers study is based on participants from two existing prospective cohort studies: EPIC-Italy [19] and the Northern Sweden Health and Disease Study (NSHDS) [20]. In both, blood samples were prospectively collected from healthy subjects at enrolment. 

EPIC-Italy is a subset of the European prospective Investigation into Cancer and nutrition. Overall, 47,749 volunteers aged 35-70 years were enrolled in five participating centres across Italy; Varese, Florence, Turin, Naples and Ragusa, between 1993 and 1998. At enrolment, all participants signed an informed consent form agreeing to provide detailed information on their dietary and life-style habits at recruitment and to have their health status followed for the rest of their lives. Standardized procedures were used to identify newly diagnosed cases of cancer based on automated linkages to cancer and mortality registries, municipal population offices and hospital discharge systems. In Naples follow up information was collected through periodic personal contact. 

The NSHDS includes participants from the Västerbotten Intervention program, the Västerbotten Mammary Screening Program and the Northern Sweden MONICA project. A total of 95,000 healthy individuals aged 40-60 were invited for inclusion in the project between 1990 and 2006. At initial recruitment, subjects were asked to complete a self- administered questionnaire to collect demographic, medical and lifestyle information and a separate self-administered food frequency questionnaire. Informed consent was obtained from all participants and a medical examination was conducted during which a blood sample was taken. Invasive cancers occurring among cohort members during the study period were identiﬁed by linkage with the Swedish Cancer Registry and the local Northern Sweden Cancer Registry.

Lymphoma cases that occurred within the two cohorts between 2 and 16 years of follow up were identified. Lymphoma cases were classified into subtypes according to the SEER ICD-0-3 morphology codes [21]. All eligible B-cell NHL cases, including multiple myeloma were included. For each case one suitable cancer-free control was selected by incidence matching from the remaining populations matched on gender, age (+/- 2.5 years), centre and date of blood collection (+/- 6 months). More than 95% of participants also had the same fasting status as their matched pair at blood extraction. Information from the two studies was integrated into a single database and calibrated. 

### Exposure Assessment

Exposure to metals was assessed by measurements in stored erythrocytes. Blood samples were collected in citrate (Italy) or heparin (Sweden) tubes and processed by centrifugation on the same day of collection (Italy) and within one hour of collection (Sweden). Aliquots (0.5ml) were stored in liquid nitrogen tanks at -196°C in Italy and -80°C in Sweden.

The concentrations of Cd and Pb in erythrocytes were determined by inductively coupled plasma-mass spectrometry (ICP-MS; Thermo X7, Thermo Elemental, Winsford, UK) in samples diluted with an alkaline solution according to Barany et al [22]. The detection limit, calculated as 3 times the standard deviation (SD) of the blank was 0.03 and 0.09 µg/L for Cd and Pb respectively. The analytical accuracy was checked against human blood reference material from Centre de Toxicologie du Quebec, International Comparison Program, Canada. The results (mean ± SD) obtained were for Cd (Lot. C0616 and C0912) 0.98 ± 0.04 and 5.0 ± 0.18 µg/L (n = 69) vs. recommended 1.0 ± 0.13 and 5.3 ± 0.43 µg/L, respectively and for Pb (Lot. L0909 and L0807) 23 ± 0.043 and 112 ± 3.9 µg/L (n = 69) vs. recommended 23 ± 1.1 and 112 ± 9.9 µg/L, respectively. All samples were prepared in duplicate and the method imprecision (calculated as the coefficient of variation for duplicate preparation measurements) was 5 and 3% for Cd and Pb respectively. 

To reduce the impact of technically-induced variation, matched pairs were analysed in the same batch on the same day. As matching was performed by sample date each case therefore had the same storage time as its matched control. 

### Statistical analysis

We conducted all analyses on the total population as well as by cohort and gender where appropriate. To take potential subtype heterogeneity into account, subtype specific analyses were conducted to the most detailed extent allowable by sample size, according to the hierarchies proposed by Morton et al [23]. The statistical significance of the differences in baseline characteristics between cases and controls were determined using the Student’s t-test and the chi-squared test for continuous and categorical outcomes respectively. Due to typically non-Gaussian distributions of erythrocyte concentrations for the two metals, these were explored by non-parametric methods. Namely cohort and gender specific analyses used ranksum test and the case-control status analyses used the Wilcoxon signed rank test.

Cases were categorised into ordered quartiles based on the distribution of Cd or Pb in the matched control population and a further binary variable was created to assess whether blood concentration exceeded levels previously identified in the literature as ‘toxic’ or ‘harmful’: >1 µg/L [7] and >100 µg/L [1] for Cd and Pb respectively. Conditional logistic regression analyses were utilised to calculate odds ratios and 95% confidence intervals accounting for the matching factors. Unconditional logistic regression using log transformed exposure levels as a continuous variable and adjusting for the matching variables was used to consider subtype heterogeneity and the effect of time between blood draw and diagnosis. All controls were included in these analyses to maximise power. The Mantel-Haenszel score test was then used to check for significant heterogeneity between the odds ratios for the four largest subtypes and between those diagnosed within five years of blood draw compared to those diagnosed more than five years after blood draw. Confounding was assessed by including additional lifestyle variables (see Table S1 in File S1) in the models and applying the likelihood ratio test to assess the relative quality of fit.

All analyses were performed using STATA 11.3 (Stata Corporation, College Station, TX).The level of statistical significance was set at 0.05 and all tests are two tailed.

## Results

A total of 270 incident cases were identified during follow-up; 84 from EPIC Italy and 186 from NSHDS, including 76 cases of multiple myeloma (see Figure S1 in File S1). There were no significant differences between cases and controls for any of the baseline variables investigated (Table 1). 

**Table 1:** Baseline characteristics of cases and controls.

Baseline variable		Case (*n*=270)	Control (*n*=270)	Difference
		[*n* (%)]	[*n* (%)]	(p-value)*
**Cohort**	**EPIC-Italy**	84 (31.1)	84 (31.1)	
	**NSHDS**	186 (68.9)	186 (68.9)	
**Gender**	**Male**	133 (49.3)	133 (49.3)	
	**Female**	137 (50.7)	137 (50.7)	
**Mean Age**	**(yrs)**	53.08	53.09	0.989
**Mean Height**	**(cm)**	169.55	168.24	0.120
**Mean Weight**	**(kg)**	76.48	75.09	0.267
**BMI**	**Underweight**	1 (0.4)	0 (0.0)	
	**Normal**	104 (38.5)	109 (40.4)	
	**Overweight**	121 (44.8)	118 (43.7)	
	**Obese**	40 (14.8)	39 (14.4)	
	**unknown**	4 (1.5)	4 (1.5)	0.883
**Smoking status**	**Never**	121 (44.8)	134 (49.6)	
	**Former**	90 (33.3)	72 (26.7)	
	**Current**	57 (21.1)	54 (20.0)	
	**unknown**	2 (0.7)	10 (3.7)	0.269
**Highest educational level**	**None**	4 (1.5)	1 (0.4)	
	**Primary**	94 (34.8)	98 (36.3)	
	**Technical/professional**	68 (25.2)	54 (20.0)	
	**Secondary**	52 (19.3)	64 (23.7)	
	**University/college**	47 (17.4)	42 (15.6)	
	**unknown**	5 (1.9)	11 (4.1)	0.202
**Cambridge physical activity index**	**Inactive**	80 (29.6)	75 (27.8)	
	**Moderately inactive**	106 (39.3)	95 (35.2)	
	**Moderately active**	69 (25.6)	76 (28.2)	
	**Active**	14 (5.2)	23 (8.5)	
	**unknown**	1 (0.4)	1 (0.4)	0.510

* P-value for difference was calculated using the chi-squared test for categorical baseline variables and the student’s t-test for continuous variables

Concentrations in all blood samples were above the limit of quantification for both metals. When the total population (cases and controls combined) was considered females had a higher body burden of Cd than males (median [range]: 0.61 μg/L [0.10, 4.32] and 0.37 μg/L [0.10, 5.22], for females and males respectively, p=0.006), while Pb was higher in males; 61.85 μg/L [15.42, 672.48] compared to females; 52.71 μg/L [11.20, 400.84] p<0.001). The median concentrations of both metals were significantly (p<0.001) higher in the Italian cohort (Cd: 0.61μg/L [0.10, 3.58] vs. 0.41μg/L [0.10, 5.22] in Sweden. Pb: 91.91μg/L [39.29, 400.84] vs. 44.99μg/L [11.20, 672.48] in Sweden). There was a weak positive correlation between erythrocyte concentrations of Cd and Pb (r^2^=0.190, p<0.001), which was evident in both cohorts. 

There was no significant difference in median or mean erythrocyte concentrations between cases and controls for either metal (tables S2 and S3 in File S1). There was also no difference when the results were stratified by cohort or gender, Tables 2 and 3 describe the risk estimates when exposure levels were categorised into quartiles based on the control population, again no significant increases were observed. In the full population the risk of B-cell NHL in the highest compared to the lowest quartile of exposure was OR 0.93 (95% CI: 0.43, 2.02) and OR 1.09 (95% CI: 0.61, 1.93) for Pb and Cd, respectively. While for multiple myeloma the odds ratios were OR 1.63 (95% CI: 0.45, 5.94) for Pb and OR 1.16 (95% CI: 0.40, 3.40) for Cd.

**Table 2:** Association between prediagnostic exposure levels of Pb by quartile and risk of B-cell NHL and Multiple myeloma.

	B-cell NHL						Multiple myeloma					
Population	Quartile (µg/L)	Cases (*n*)	Controls (*n*)	OR^a^ (95% CI)	P-value	*p for trend*	Quartile (µg/L)	Cases (*n*)	Controls (*n*)	OR^a^ (95% CI)	P-value	*p for trend*
**Total**	Q1 (15.423, 39.286)	51	48	1			Q1 (11.199, 35.133)	17	19	1		
	Q2 (39.504, 58.763)	48	49	0.93 (0.51, 1.67)	0.799		Q2 (35.184, 51.973)	20	19	1.30 (0.44, 3.86)	0.636	
	Q3 (58.832, 87.218)	47	48	0.91 (0.47, 1.79)	0.792		Q3 (52.459, 79.079)	17	19	1.17 (0.38, 3.59)	0.781	
	Q4 (87.531, 400.843)	48	48	0.93 (0.43, 2.02)	0.852	0.849	Q4 (81.448, 672.482)	22	19	1.63 (0.45, 5.94)	0.459	0.533
**Sweden**	Q1 (15.423, 35.819)	42	32	1			Q1 (11.199, 30.604)	17	14	1		
	Q2 (35.948, 46.842)	29	33	0.61 (0.28, 1.33)	0.212		Q2 (31.563, 40.417)	6	13	0.41 (0.11, 1.45)	0.165	
	Q3 (47.114, 60.969)	24	32	0.53 (0.24, 1.16)	0.111		Q3 (41.463, 56.314)	14	14	0.74 (0.22, 2.50)	0.634	
	Q4 (61.338, 244.382)	36	33	0.73 (0.33, 1.62)	0.441	0.488	Q4 (57.597, 672.482)	18	14	0.94 (0.28, 3.10)	0.914	0.644
**Italy**	Q1 (39.286, 71.980)	18	16	1			Q1 (48.623, 81.448)	7	6	1		
	Q2 (72.363, 89.503)	12	15	0.74 (0.29, 1.93)	0.543		Q2 (83.455, 92.973)	4	5	0.70 (0.13, 3.87)	0.682	
	Q3 (89.575, 125.327)	18	17	0.96 (0.35, 2.61)	0.938		Q3 (93.128, 137.177)	6	5	1.00 (0.21, 4.71)	1	
	Q4 (133.650, 400.443)	15	15	0.91 (0.32, 2.56)	0.86	0.934	Q4 (141.978, 228.143)	4	5	0.62 (0.09, 4.49)	0.636	0.768
**Males**	Q1 (15.423, 44.989)	30	24	1			Q1 (19.898, 36.049)	5	9	∞		
	Q2 (45.444, 61.498)	18	25	0.57 (0.23, 1.37)	0.208		Q2 (38.613, 52.578)	8	8			
	Q3 (61.904, 100.201)	28	25	0.83 (0.35, 1.99)	0.678		Q3 (52.808, 93.128)	14	8			
	Q4 (100.528, 378.943)	23	24	0.74 (0.27, 2.04)	0.558	0.742	Q4 (97.683, 672.482)	7	9			
**Females**	Q1 (17.019, 36.079)	29	23	1			Q1 (11.199, 30.604)	12	10	1		
	Q2 (36.719, 54.739)	23	24	0.62 (0.23, 1.65)	0.337		Q2 (32.928, 48.623)	10	11	0.71 (0.20, 2.57)	0.607	
	Q3 (55.401, 77.823)	22	24	0.54 (0.20, 1.46)	0.225		Q3 (49.859, 75.424)	10	11	0.71 (0.19, 2.61)	0.606	
	Q4 (78.313, 400.843)	21	24	0.42 (0.12, 1.47)	0.174	0.17	Q4 (76.344, 220.943)	10	10	0.74 (0.14, 3.83)	0.715	0.692

^a^ Risk of NHL by exposure quartile compared to the lowest quartile (ref). Quartiles based on exposure distribution in controls. Computed using conditional logistic regression

* p<0.05

∞ Insufficient numbers for model

**Table 3:** Association between prediagnostic exposure levels of Cd and risk of NHL.

	B-cell NHL						Multiple myeloma					
Population	Quartile (µg/L)	Cases (*n*)	Controls (*n*)	OR^a^ (95% CI)	P-value	*p for trend*	Quartile (µg/L)	Cases (*n*)	Controls (*n*)	OR^a^ (95% CI)	*P-value*	*p for trend*
**Total**	Q1 (0.143, 0.316)	55	48	1			Q1 (0.098, 0.299)	17	19	1		
	Q2 (0.318, 0.498)	32	49	0.55 (0.30, 1.04)	0.066		Q2 (0.305, 0.495)	19	19	1.14 (0.42, 3.12)	0.787	
	Q3 (0.498, 0.738)	48	48	0.86 (0.47, 1.60)	0.64		Q3 (0.498, 0.938)	21	19	1.25 (0.49, 3.17)	0.637	
	Q4 (0.741, 5.224)	59	48	1.09 (0.61, 1.93)	0.776	0.461	Q4 (0.973, 4.113)	19	19	1.16 (0.40, 3.40)	0.789	0.744
**Sweden**	Q1 (0.143, 0.268)	32	32	1			Q1 (0.098, 0.234)	13	13	1		
	Q2 (0.269, 0.400)	29	33	0.93 (0.46, 1.87)	0.831		Q2 (0.238, 0.418)	17	14	1.16 (0.36, 3.80)	0.803	
	Q3 (0.404, 0.669)	32	32	1.06 (0.50, 2.27)	0.871		Q3 (0.419, 0.883)	13	14	0.95 (0.30, 2.96)	0.924	
	Q4 (0.670, 5.224)	38	33	1.20 (0.59, 2.45)	0.614	0.527	Q4 (0.973, 4.113)	12	14	0.83 (0.21, 3.25)	0.791	0.623
**Italy**	Q1 (0.208, 0.380)	13	15	1			Q1 (0.178, 0.401)	1	5	1		
	Q2 (0.383, 0.556)	11	17	0.54 (0.17, 1.68)	0.286		Q2 (0.461, 0.691)	11	5	5.40 (0.58, 50.95)	0.137	
	Q3 (0.563, 0.800)	18	17	1.25 (0.46, 3.41)	0.666		Q3 (0.713, 0.938)	2	6	1.00 (0.05, 20.89)	1	
	Q4 (0.818, 3.586)	21	14	2.09 (0.74, 5.91)	0.163	0.175	Q1 (1.083, 2.801)	7	5	4.27 (0.39, 46.81)	0.234	0.549
**Males**	Q1 (0.143, 0.258)	29	24	1			Q1 (0.099, 0.195)	6	8	1		
	Q2 (0.259, 0.373)	22	25	0.73 90.33, 1.60)	0.433		Q2 (0.208, 0.461)	16	9	3.20 (0.64, 16.02)	0.156	
	Q3 (0.378, 0.800)	28	25	0.98 (0.46, 2.08)	0.948		Q3 (0.468, 0.765)	6	9	1.00 (0.24, 4.20)	0.995	
	Q4 (0.803, 5.224)	20	24	0.65 (0.27, 1.56)	0.333	0.563	Q4 (0.778, 3.784)	6	8	0.84 (0.11, 6.62)	0.871	0.495
**Females**	Q1 (0.185, 0.375)	19	23	1			Q1 (0.098, 0.383)	10	10	1		
	Q2 (0.380, 0.556)	15	24	0.62 (0.23, 1.64)	0.332		Q2 (0.394, 0.495)	2	11	0.12 (0.01, 1.10)	0.061	
	Q3 (0.559, 0.719)	24	25	1.21 (0.50, 2.91)	0.671		Q3 (0.498, 1.083)	15	10	1.28 (0.37, 4.46)	0.697	
	Q4 (0.738, 4.324)	37	23	1.95 (0.87, 4.37)	0.106	0.052	Q4 (1.214, 4.113)	15	11	1.23 (0.39, 3.94)	0.725	0.251

^a^ Risk of NHL by exposure quartile compared to the lowest quartile (ref). Quartiles based on exposure distribution in controls. Computed using conditional logistic regression

* p<0.05

Information on histological subtype was available for 225 of 270 cases (83%) and the remaining 45 cases were classified as ‘B-cell lymphoma not otherwise specified’. There was little evidence of an increased risk associated with metal exposure for any of the four largest subtype groups; multiple myeloma, diffuse large B-cell lymphoma, B-cell chronic lymphatic leukaemia, or follicular lymphoma, although there was a consistent non-significant increase in risk in females across all subtypes(Tables 4 and 5 for Pb and Cd respectively). Despite apparent heterogeneous estimates of odds ratios across the subtypes (in every population of interest), Mantel-Haenszel score did reveal any significant differences results (p>0.3).

**Table 4:** Association between prediagnostic exposure levels of Pb and risk of NHL by subtype.

Subtype	Population	Cases		Controls^a^			
		*n*	median (µg/L)	*n*	median (µg/L)	OR^b^ (95% CI)	P-value
**Multiple Myeloma**	**Total**	76	55.338	269	57.597	1.04 (0.57, 1.90)	0.888
	**Sweden**	55	44.674	185	45.464	1.23 (0.64, 2.37)	0.538
	**Italy**	21	92.373	84	89.594	0.33 (0.07, 1.59)	0.168
	**Men**	34	66.320	132	60.926	0.83 (0.35, 1.96)	0.676
	**Women**	42	47.958	137	53.407	1.28 (0.53, 3.08)	0.587
**Diffuse Large B-cell Lymphoma**	**Total**	45	53.467	269	57.597	0.60 (0.26, 1.40)	0.239
	**Sweden**	34	48.576	185	45.464	0.70 (0.28, 1.73)	0.433
	**Italy**	11	100.328	84	89.594	0.38 (0.03, 5.04)	0.459
	**Men**	24	61.473	132	60.926	0.97 (0.35, 2.64)	0.949
	**Women**	21	53.294	137	53.407	0.29 (0.07, 1.18)	0.085
**B-cell Chronic Lymphatic Lymphoma**	**Total**	42	51.392	269	57.597	0.71 (0.32, 1.57)	0.392
	**Sweden**	31	40.927	185	45.464	1.00 (0.43, 2.33)	0.996
	**Italy**	11	65.845	84	89.594	0.12 (0.02, 0.87)	0.036*
	**Men**	25	55.933	132	60.926	0.63 (0.23, 1.74)	0.376
	**Women**	17	44.373	137	53.407	0.79 (0.17, 3.60)	0.764
**Follicular Lymphoma**	**Total**	39	66.968	269	57.597	1.17 (0.52, 2.63)	0.701
	**Sweden**	19	44.157	185	45.464	1.19 (0.43, 3.29)	0.738
	**Italy**	20	90.278	84	89.594	1.14 (0.30, 4.31)	0.850
	**Men**	19	78.363	132	60.926	0.80 (0.25, 2.55)	0.706
	**Women**	20	66.479	137	53.407	1.91 (0.54, 6.78)	0.317

^a^ For each subpopulation (Sweden, Italy, Males, Females) all controls were used, regardless of case subtype, in order to maximise power

^b^ Risk of NHL associated with a one unit increase in log transformed exposure levels. Computed using unconditional logistic regression adjusting for sex, age, centre, batch and sample date

* p<0.05

**Table 5:** Association between prediagnostic exposure levels of Cd and risk of NHL by subtype.

Subtype	Population	Cases		Controls^a^			
		*n*	median (µg/L)	*n*	median (µg/L)	OR^b^ (95% CI)	P-value
**Multiple Myeloma**	**Total**	76	0.531	269	0.498	1.07 (0.77, 1.49)	0.683
	**Sweden**	55	0.383	185	0.410	0.92 (0.63, 1.34)	0.664
	**Italy**	21	0.603	84	0.589	2.34 (0.89, 6.12)	0.084
	**Men**	34	0.346	132	0.384	0.73 (0.43, 1.24)	0.248
	**Women**	42	0.662	137	0.543	1.51 (0.92, 2.46)	0.102
**Diffuse Large B-cell Lymphoma**	**Total**	45	0.539	269	0.498	0.95 (0.61, 1.48)	0.804
	**Sweden**	34	0.435	185	0.410	0.92 (0.57, 1.49)	0.730
	**Italy**	11	0.633	84	0.589	1.50 (0.42, 5.33)	0.529
	**Men**	24	0.362	132	0.384	0.91 (0.51, 1.63)	0.755
	**Women**	21	0.638	137	0.543	1.13 (0.53, 2.39)	0.754
**B-cell Chronic Lymphatic Lymphoma**	**Total**	42	0.398	269	0.498	0.82 (0.52, 1.30)	0.400
	**Sweden**	31	0.370	185	0.410	0.74 (0.43, 1.25)	0.256
	**Italy**	11	0.904	84	0.589	1.04 (0.34, 3.18)	0.950
	**Men**	25	0.299	132	0.384	0.45 (0.21, 0.97)	0.040*
	**Women**	17	0.618	137	0.543	1.48 (0.73, 3.00)	0.271
**Follicular Lymphoma**	**Total**	39	0.598	269	0.498	1.48 (0.94, 2.32)	0.088
	**Sweden**	19	0.568	185	0.410	1.27 (0.74, 2.20)	0.389
	**Italy**	20	0.606	84	0.589	2.83 (1.11, 7.20)	0.029*
	**Men**	19	0.538	132	0.384	1.52 (0.87, 2.67)	0.141
	**Women**	20	0.599	137	0.543	1.51 (0.70, 3.25)	0.293

^a^ For each subpopulation (Sweden, Italy, Males, Females) all controls were used, regardless of case subtype, in order to maximise power

^b^ Risk of NHL associated with a one unit increased in log transformed exposure levels. Computed using unconditional logistic regression adjusting for sex, age, centre, batch and sample date

* p<0.05

The number and percentage of cases and controls above the toxic or harmful concentration level for Pb and Cd are shown in tables 6 and 7 respectively. There was no significantly increased risk of B-cell NHL or of multiple myeloma associated with being above 100µg/L for Pb. (Table 6). .Females above 1µg/L for Cd had more than double the risk of B-cell NHL compared to those in the lowest quartile of exposure (OR 2.20 95%CI; 1.04, 4.65) (Table 7). An increased risk was also observed for multiple myeloma, although it was not significant (OR; 1.43 95%CI 0.54, 3.75). 

**Table 6:** Number (%) of cases and controls above toxic levels of Pb (>100µg/L) and association with NHL.

Subtype	Population	Cases n. (%)	Controls n. (%)	OR^a^ (95% CI)	P-value
**B-cell NHL**	**Total**	*37 (19.1)*	*35 (18.0)*	1.10 (0.60, 2.02)	0.758
	**Sweden**	*10 (7.6)*	*8 (6.1)*	1.29 (0.48, 3.45)	0.618
	**Italy**	*27 (42.9)*	*27 (42.9)*	1.00 (0.46, 2.16)	1.000
	**Men**	*24 (24.2)*	*25 (25.3)*	0.93 (0.44, 1.98)	0.847
	**Women**	*13 (13.7)*	*10 (10.5)*	1.50 (0.53, 4.21)	0.442
**Multiple myeloma**	**Total**	*13 (17.1)*	*11 (14.5)*	1.29 (0.48, 3.45)	0.618
	**Sweden**	*5 (9.1)*	*2 (3.6)*	2.50 (0.49,12.89)	0.273
	**Italy**	*8 (38.1)*	*9 (42.9)*	0.80 (0.21, 2.98)	0.739
	**Men**	*7 (20.6)*	*8 (23.5)*	0.80 (0.21, 2.98)	0.739
	**Women**	*6 (14.3)*	*3 (7.1)*	2.50 (0.49, 12.89)	0.273

^a^ Risk of NHL in those above the toxic level of exposure compared to the risk of NHL in those below the toxic level of exposure. Computed using conditional logistic regression

* p<0.05

**Table 7:** Number (%) of cases and controls above toxic levels of Cd (>1µg/L) and association with NHL.

Subtype	Population	Cases n. (%)	Controls n. (%)	OR^a^ (95% CI)	P-value
**B-cell NHL**	**Total**	*39 (20.1)*	*30 (15.5)*	1.39 (0.81, 2.38)	0.227
	**Sweden**	*24 (18.3)*	*22 (16.8)*	1.11 (0.59, 2.10)	0.746
	**Italy**	*15 (23.8)*	*8 (12.7)*	2.40 (0.85, 6.81)	0.100
	**Men**	*14 (14.1)*	*17 (17.2)*	0.77 (0.34, 1.75)	0.533
	**Women**	*25 (26.3)*	*13 (13.7)*	2.20 (1.04, 4.65)	0.039*
**Multiple myeloma**	**Total**	*19 (25.0)*	*18 (23.7)*	1.09 (0.48, 2.47)	0.835
	**Sweden**	*12 (21.8)*	*13 (23.6)*	0.88 (0.32, 2.41)	0.796
	**Italy**	*7 (33.3)*	*5 (23.8)*	1.67 (0.40, 6.97)	0.484
	**Men**	*4 (11.8)*	*6 (17.6)*	0.50 (0.09, 2.73)	0.423
	**Women**	*15 (35.7)*	*12 (28.6)*	1.43 (0.54, 3.75)	0.469

^a^ Risk of NHL in those above the toxic level of exposure compared to the risk of NHL in those below the toxic level of exposure. Computed using conditional logistic regression

* p<0.05

The median time from blood collection to diagnosis was 6.6 years in NSHDS and 5.0 in Epic-Italy (table S4 in File S1). There was no association between time from blood draw to NHL diagnosis and erythrocyte concentrations of Cd (p=0.555) or Pb (p=0.549) in the cases. Although there did appear to be some differences between cases with ≥ 5 years between blood draw and diagnosis compared to those with ≤ 5 years, in certain subpopulations (table S5 in File S1) there was no significant heterogeneity in the odds ratios (Mantel-Haenzsel score test p<0.5 for all subpopulations). 

 Adjustment of these models for potential confounders did not alter any of the findings (results not shown). 

## Discussion

Pb and Cd are two of the most widespread environmental and industrial toxins globally [10] and they have both been associated with a range of medical conditions [2,4,24,25] .Whilst Cd is a known human carcinogen, the evidence for Pb is less compelling. We conducted a prospective study to determine whether these two metals were associated with an increased risk of NHL, which represents the first such study in this field. Our results indicate an association between B-cell NHL and Cd in females, particularly at the relatively high exposure levels defined as toxic or harmful in this study. However no such relationship was evident in males. Similarly we did not observe an association between prediagnostic levels of Pb and risk of NHL in either gender. 

Cohort and gender-specific differences were evident throughout the study; Cd was found to be at higher concentrations in females and in the Italian cohorts, and it was in these subgroups that the strongest associations with B-cell NHL were observed. There was additionally some evidence that risk may differ by subtype, however, the subtype analyses were limited by the small number of observations. Additional analysis not presented in the manuscript did not provide any evidence for confounding in the relationship between either metal and B-cell NHL.

Metals may induce carcinogenesis through a number of mechanisms. In vitro studies indicate that both Cd and Pb have genotoxic properties [10,11] and there is evidence that both metals can induce DNA damage via elevated levels of oxidative stress [1]. However, although the evidence from animal models is strong, epidemiological evidence in humans, particularly for Pb, is less consistent and lacks a clear dose–response relationship [11,26]. Studies in humans have mainly been based on small, male, occupationally exposed populations and tend to lack sufficient information on historical exposure [7,26]. Furthermore the strongest epidemiological evidence for Pb comes from studies of population living near to oil refineries, which also emit other suspected haematological carcinogens such as benzene and dioxins [14,27]. 

In our study of a general, non-occupationally exposed cohort, exposure was assessed using erythrocyte concentrations of Pb and Cd in blood samples taken from cancer-free individuals at recruitment to the cohort. This provides a point estimate of historical exposure free from recall bias. Although urine is considered to be the best biomaterial to measure biomarkers of lifetime Cd exposure, blood Cd has been found to correlate highly with Cd concentrations in both urines and tissues and as such can be considered as a good estimate of body burden [6]. Pb binds to erythrocytes and therefore body burden is optimally assessed by blood concentration [24]. Exposure levels for both metals were within the range of those reported in studies of similar populations [25,28,29]. Our finding of higher levels in females is consistent with previous studies [30] and is thought to be related to the low iron stores common in women of fertile age [29]. Similarly we observed higher Pb levels in males as expected [30].

Despite evidence from animal studies we could find only one previous study which directly explored the association between Cd and NHL in humans; Adams et al [31] reported an association between urinary Cd and NHL in males and an association with leukaemia in both males and females, a finding consistent with a case control study in a Turkish population [17,31]. To the best of our knowledge, our study is the first to report on the risk of any haematological malignancy and Pb exposure using measures of body burden.

The biological rationale for the risk effects of Cd and Pb is based partly on their interaction with the immune system. Cd may act through mechanisms related to the increase of inflammatory cytokines [15,32]. While Pb has been reported to decrease the absolute number and percentage of CD8^+^ and CD4^+^ cells [3,6,33]; such a decrease has been previously associated with well characterised NHL risk factors including Trichloroethylene and Carbofuran exposure [34–37]. Interestingly Pb exposure has also been associated with Ig-E mediated type1 hypersensitivity in some studies and it has been suggested it may contribute to an increase in allergic conditions [16]. Atopy and allergy have been reported to reduce the risk of NHL [12] and therefore this may be affecting our results.

Metal-induced carcinogenesis is dependent on the route, level, and duration of exposure which is subject to individual variation. However, even when exposed at the same levels, host factors such as medical history, smoking habits and simultaneous exposure to other toxic agents may play an important role in determining the individual burden of both metals [2]. Notably a number of studies have reported on genetic polymorphisms which influence the metabolism and storage of these metals and which may affect our results. Large individual variation in the expression of Metallothionein, a protein which binds the majority of Cd in the body [1] and polymorphisms in SLC4A7 and ALAD, which affect Pb influx and the binding of Pb in erythrocytes [38,39], have been identified as candidates for the variation in concentrations of these metals between individuals. 

The gender differences observed in our findings are of particular note, given the recent interest in gender-specific susceptibility to carcinogens [40]. A growing body of mechanistic, experimental and epidemiological evidence suggests that women may be more susceptible to the effect of carcinogens than males [41]. This relates both to baseline differences in exposure levels to putative carcinogens as well hormonal, physiological, genetic and epigenetic effects[42]. Gender-differences in the expression of xenobiotic metabolising enzymes including the cytochrome P450 system and the human glutathione S-transferases [41,42], and in candidate transporter genes for carcinogen export have been reported[40]. While a number of studies have observed greater levels of markers of DNA damage including DNA adduct levels, sister chromatid exchanges (SCEs) and micronucleus frequency in female cancer patients compared to similarly exposed male cases [40,42]. DNA repair capacity has also been reported to be substantially lower in females, while expression of the oncogenic k-ras mutation has been found to be higher. It is thought that these effects may be regulated by hormonal responses and that estrogen in particular may play an important role [40–42].

Alternatively our findings may simply reflect the lower baseline risk of NHL in unexposed women[40]; NHL is 50% more common among men [43]. However, other Cd-associated health effects have previously been observed to be more common in women than men [30] and we believe our results indicate the need for further gender-specific studies. 

Subtype-specific analyses, to the most detailed extent possible within the constraints of our sample size, were conducted in addition to the analysis of the total population. Although also a B-cell derived malignancy, due to its pathophysiological differences to the other subtypes multiple myeloma has been evaluated separately in this manuscript. We observed no significant heterogeneity between subtypes. Aetiology is largely unknown for most B-cell NHL subtypes and although there is evidence that some risk factors are subtype specific, others such as benzene [44], have been found to contribute to multiple subtypes. It is of note that, in this study our only significant finding; that of an increased risk with Cd exposure in females, was evident, although non-significant, across all subtypes including multiple myeloma. The lack of significance in the subtype-specific analyses may simply reflect the small sample size. 

Previous studies of NHL and environmental exposures have noted an effect of ‘time to diagnosis’ i.e. the time between sample collection and diagnosis date; with stronger associations observed closer to diagnosis [45–47]. This is thought to be due to the pathophysiologic changes such as lipid mobilization, weight loss, and metabolic changes, accompanying carcinogenesis which may affect the body burden of exposures [48]. To ensure that pre-diagnostic subclinical changes in cases who were diagnosed shortly after blood draw were not modifying the body burden of exogenous exposures [48] we stratified on the time between blood draw and diagnosis. The association with Cd was fairly consistent across strata although there was some indication that the findings in subset analyses of women may have been driven by the individuals with <5 years from sampling to diagnosis, based on the larger effect size in this group.Conversely, the results suggested a more strongly inverse association with Pb in those who were diagnosed less than five years after blood draw, which may be the result of lowered Pb concentrations due to subclinical cancer. However these analyses were limited by small numbers and deserve replication in a larger cohort with sufficient power.

There were a number of strengths of this study; our prospective study design protected against selection bias and the problem of reverse causation. Our case and control populations were similar with respect to baseline factors and additional confounders. Finally we attempted to exclude from some analyses participants whose body burden of metals may have been influenced by pre-cancerous changes. Conversely our study lacked information on family history of NHL and on grade or staging of the tumours. Additionally, we were not able to control for other unmeasured metals and trace elements which may be exerting interaction effects on the risk of NHL. We only have a one spot measurement so our exposure estimate is based on a single time period that is not necessarily representative of lifetime exposure.

## Conclusions

In conclusion in this nested case control study we do not find a consistent association with prediagnostic erythrocyte concentrations of Cd or Pb and risk of NHL, although there is some indication that high exposure to Cd may increase risk in females only. Our results were robust to adjustment and do not seem to be the result of confounding, although we did not correct for multiple testing and cannot rule out the possibility of false positives. Our findings may suggest that the carcinogenic potential of Cd is only realised at high concentrations and therefore levels in males may have been too low to show a clinical effect. This could also explain the differing results by cohort: Italy, in which levels of Cd were higher than in Sweden, showed the greatest risk. However gender-specific susceptibility to carcinogens is also likely to play a role. We report an essentially null association between B-cell NHL risk and prediagnostic concentrations of Pb. 

These results are among the first in the field of metal exposure and NHL aetiology and although there is little to suggest that Cd or Pb constitute major risk factors for NHL, Cd exposure in females in particular is worthy of further investigation. Our findings underline the need for a larger sample size and the importance of gender-specific analyses in any such future studies. 

## Supporting Information

File S1 **Table S1.** Questionnaire variables considered as potential confounders. **Figure S1.** Flow chart of participation. **Table S2.** Mean, median and range of Pb erythrocyte concentrations (µg/L) in cases and controls. **Table S3.** Mean, median and range of Cd erythrocyte concentrations (µg/L) in cases and controls. **Table S4.** Time between sample extraction and NHL diagnosis; mean median, range and number of cases by 5 year interval. **Table S5.** Risk of B-cell NHL with pre-diagnostic exposure levels of Pb and Cd stratified by time to diagnosis
(DOCX)
