# Supplementary material for: Blood Erythrocyte Concentrations of Cadmium and Lead and the Risk of B-Cell Non-Hodgkin’s Lymphoma and Multiple Myeloma: A Nested Case-Control Study
Source: PLoS One. 2013 Nov 28;8(11):e81892. doi: 10.1371/journal.pone.0081892 (PMC3842971; doi:10.1371/journal.pone.0081892)
Supplement: File S1 — Table S1. Questionnaire variables considered as potential confounders. Figure S1. Flow chart of participation. Table S2. Mean, median and range of Pb erythrocyte concentrations (µg/L) in cases and controls. Table S3. Mean, median and range of Cd erythrocyte concentrations (µg/L) in cases and controls. Table S4. Time between sample extraction and NHL diagnosis; mean median, range and number of cases by 5 year interval. Table S5. Risk of B-cell NHL with pre-diagnostic exposure levels of Pb and Cd stratified by time to diagnosis (DOCX) [file pone.0081892.s001.docx]

**Supplementary material: Blood Erythrocyte Concentrations of Cadmium and Lead and the Risk of B-cell Non-Hodgkin’s Lymphoma and Multiple Myeloma: A Case-control Study**

*Rachel S Kelly, Thomas Lundh, Miquel Porta, Ingvar A. Bergdahl, Domenico Palli, Ann-Sofie Johansson, Maria Botsivali, Paolo Vineis, Roel Vermeulen, Soterios A Kyrtopoulos and Marc Chadeau-Hyam on behalf of the EnviroGenoMarkers project consortium*

**Table S1: Questionnaire variables considered as potential confounders**

Subset of selected variables from full cohort questionnaire

EPIC-Italy: *see* <http://epic.iarc.fr/research/quest.php>

NSHDS: *see* <http://www.biobanks.se/medicalbiobank.htm>

|  | **Question** | |
| --- | --- | --- |
| **Variable** | **EPIC-Italy** | **NSHDS** |
| **Age** | Age at sampling | Age at sampling |
| **Height (cm)** | Height [*measured*] | Height in cm [*measured in relation to sampling with the exception of participants in the mammography cohort sampled prior to September 1998 who self-reported*] |
| **Weight (kg)** | Weight [*measured*] | Weight in kg [*measured in relation to sampling with the exception of participants in the mammography cohort sampled prior to September 1998 who self-reported*] |
| **BMI (kg/m^2^)** | BMI [*computed*] | BMI [*computed*] |
| **Smoking status** | Do you smoke cigarettes?   1. = Currently 2. = Previously 3. = Never | Smoking status:  N = Non-smoker F = Former smoker C= Current smoker |
| **Highest educational level** | What was your highest school/college/university level?   1. = None 2. = primary education 3. = lower secondary   education   1. = Vocational school 2. = Diploma 3. = Community college 4. = Degree | What was your highest level of (completed) education?   1. = Elementary school + nine year   (compulsory) school   1. = Folk high school equivalent to   nine-year (compulsory)  school+ junior secondary school  + girls’ school + vocational  (training) school   1. = Folk high school equivalent to   upper secondary school + girls’  school equivalent to upper  secondary school  education   1. = University education/College |
| **Cambridge physical activity index** | *Computed from multiple indices of physical activity*   1. = Inactive 2. = Moderately inactive 3. = Moderately active   4 = Active | *Computed from multiple indices of physical activity*   1. = Inactive 2. = Moderately inactive 3. = Moderately active 4. = Active |

**Figure S1: Flow chart of participation**

**Included controls**

EPIC-Italy: 84

NSHDS: 186

***Matching***

*Sex age centre date of blood draw*

**Total number of B-cell NHL cases**

EPIC-Italy: 189

NSHDS: 294

**Cancer-free controls**

**Included cases**

EPIC-Italy: 84

NSHDS: 186

**Total number of incident lymphoma cases diagnosed between 1993 and 2007**

EPIC-Italy: 215

NSHDS: 302

**Total number of Participants**

EPIC-Italy: 47,749

NSHDS: 83,000

**Excluded cases**

***-No matched control -Time between blood draw and diagnosis<2yrs -Questionnaire/blood sample missing***

EPIC-Italy: 105

NSHDS: 108

**Table S2; Mean, median and range of Pb erythrocyte concentrations (µg/L) in cases and controls**

| **Subtype** | **Population** | **Cases** | | | | **Controls** | | | | **Difference** |
| --- | --- | --- | --- | --- | --- | --- | --- | --- | --- | --- |
|  |  | ***n*** | **Median** | **Mean** | **Range** | ***n*** | **Median** | **Mean** | **Range** | **p-value^a^** |
| **Bcell NHL** | **Total** | 194 | 58.097 | 70.513 | (15.423, 400.843) | 193 | 58.763 | 71.884 | (17.414, 378.943) | 0.750 |
|  | **Sweden** | 131 | 44.373 | 53.533 | (15.423, 244.382) | 130 | 46.991 | 54.115 | (17.414, 232.383) | 0.874 |
|  | **Italy** | 63 | 93.018 | 105.820 | (48.478, 400.843) | 63 | 89.575 | 108.550 | (39.286, 378.943) | 0.784 |
|  | **Men** | 99 | 63.484 | 76.829 | (15.423, 244.282) | 98 | 61.701 | 82.881 | (26.328, 378.943) | 0.763 |
|  | **Women** | 95 | 53.294 | 63.930 | (17.019, 400.843) | 95 | 55.401 | 60.540 | (17.414, 171.68) | 0.796 |
| **Multiple Myeloma** | **Total** | 76 | 55.338 | 70.681 | (16.828, 342.782) | 76 | 52.216 | 72.855 | (11.199, 672.482) | 0.988 |
|  | **Sweden** | 55 | 44.674 | 59.197 | (16.828, 342.782) | 55 | 41.463 | 56.227 | (11.199, 672.482) | 0.291 |
|  | **Italy** | 21 | 92.373 | 100.759 | (48.623, 204.793) | 21 | 92.973 | 116.407 | (52.808, 228.143) | 0.099 |
|  | **Men** | 34 | 66.320 | 83.280 | (19.898, 342.782) | 34 | 52.693 | 91.997 | (24.322, 672.482) | 0.925 |
|  | **Women** | 42 | 47.958 | 60.482 | (16.828, 160.993) | 42 | 49.006 | 57.360 | (11.199, 220.943) | 0.955 |

^a^Differences between median concentrations in cases and controls according to the Wilcoxon signed-rank test for paired samples

**Table S3; Mean, median and range of Cd erythrocyte concentrations (µg/L) in cases and controls**

| **Subtype** | **Population** | **Cases** | | | | **Controls** | | | | **Difference** |
| --- | --- | --- | --- | --- | --- | --- | --- | --- | --- | --- |
|  |  | ***n*** | **Median** | **Mean** | **Range** | ***n*** | **Median** | **Mean** | **Range** | **p-value^a^** |
| **Bcell NHL** | **Total** | 194 | 0.543 | 0.728 | (0.143, 3.599) | 193 | 0.498 | 0.739 | (0.150, 5.224) | 0.406 |
|  | **Sweden** | 131 | 0.423 | 0.682 | (0.143, 3.599) | 130 | 0.403 | 0.767 | (0.150, 5.224) | 0.937 |
|  | **Italy** | 63 | 0.633 | 0.825 | (0.223, 3.586) | 63 | 0.556 | 0.681 | (0.208, 2.758) | 0.161 |
|  | **Men** | 99 | 0.364 | 0.609 | (0.143, 3.093) | 98 | 0.373 | 0.743 | (0.150, 5.224) | 0.486 |
|  | **Women** | 95 | 0.635 | 0.852 | (0.185, 3.599) | 95 | 0.559 | 0.734 | (0.205, 4.324) | 0.070 |
| **Multiple Myeloma** | **Total** | 76 | 0.531 | 0.839 | (0.098, 4.113) | 76 | 0.498 | 0.739 | (0.099, 3.903) | 0.768 |
|  | **Sweden** | 55 | 0.383 | 0.795 | (0.0978, 4.113) | 55 | 0.403 | 0.767 | (0.099, 3.903) | 0.940 |
|  | **Italy** | 21 | 0.603 | 0.955 | (0.379, 2.801) | 21 | 0.556 | 0.681 | (0.178, 1,821) | 0.434 |
|  | **Men** | 34 | 0.346 | 0.611 | (0.128, 3.784) | 34 | 0.373 | 0.743 | (0.099, 3.079) | 0.437 |
|  | **Women** | 42 | 0.662 | 1.024 | (0.098, 4.113) | 42 | 0.559 | 0.734 | (0.179, 3.903) | 0.385 |

^a^Differences between median concentrations in cases and controls according to the Wilcoxon signed-rank test for paired samples

*p<0.05

**Table S4; Time between sample extraction and NHL diagnosis; mean median, range and number of cases by 5 year interval**

| **Population** | **Median (months)** | **Mean (months)** | **Range** | ***n*. <5 years (<60 months)** | ***n*. ≥ 5 years ( ≥ 60 months)** |
| --- | --- | --- | --- | --- | --- |
| **Total** | 70.13 | 73.41 | (24.03, 191.08) | 121 | 149 |
| **Sweden** | 74.52 | 78.43 | (24.03, 191.08) | 70 | 116 |
| **Italy** | 54.62 | 60.25 | (24.33, 119.15) | 51 | 33 |
| **Men** | 66.16 | 73.70 | (24.33, 191.08) | 62 | 71 |
| **Women** | 71.74 | 73.11 | (24.03, 190.92) | 59 | 78 |

**Table S5; Risk of B-cell NHL with pre-diagnostic exposure levels of Pb and Cd stratified by time to diagnosis**

| **Subtype** | **Population** | **Pb (µg/L)** | | | | **Cd (µg/L)** | | | |
| --- | --- | --- | --- | --- | --- | --- | --- | --- | --- |
|  |  | **<5 years** | | **≥ 5 years** | | **<5 years** | | **≥ 5 years** | |
|  |  | **OR (95% CI)** | **p- value** | **OR (95% CI)** | **p- value** | **OR (95% CI)** | **p- value** | **OR (95% CI)** | **p- value** |
| **B cell NHL** | **Total** | 0.51 (0.24, 1.07) | 0.076 | 0.75 (0.41, 1.36) | 0.342 | 1.04 (0.70, 1.53) | 0.853 | 1.07 (0.77, 1.50) | 0.685 |
|  | **Sweden** | 0.55 (0.22, 1.35) | 0.190 | 0.78 (0.40, 1.52) | 0.465 | 0.89 (0.55, 1.42) | 0.616 | 1.04 (0.72, 1.50) | 0.840 |
|  | **Italy** | 0.41 (0.10, 1.67) | 0.216 | 0.62 (0.16, 2.3) | 0.478 | 1.45 (0.62, 3.35) | 0.388 | 1.39 (0.55, 3.48) | 0.484 |
|  | **Men** | 0.33 (0.11, 0.96) | 0.041* | 0.95 (0.45, 2.01) | 0.903 | 0.80 (0.46, 1.38) | 0.418 | 0.95 (0.62, 1.48) | 0.830 |
|  | **Women** | 0.88 (0.27, 2.89) | 0.834 | 0.50 (0.18, 1.38) | 0.180 | 1.53 (0.80, 2.96) | 0.200 | 1.32 (0.76, 2.28) | 0.322 |
| **Multiple Myeloma** | **Total** | 1.02 (0.41, 2.52) | 0.969 | 1.35 (0.59, 3.10) | 0.479 | 1.17 (0.66, 2.06) | 0.586 | 0.93 (0.57, 1.51) | 0.760 |
|  | **Sweden** | 1.36 (0.52, 3.53) | 0.532 | 1.43 (0.60, 3.37) | 0.419 | 1.05 (0.55, 2.00) | 0.881 | 0.88 (0.53, 1.48) | 0.635 |
|  | **Italy** | 0.14 (0.01, 2.83) | 0.198 | 0.70 (0.02, 31.07) | 0.855 | 1.63 (0.40, 6.60) | 0.492 | 5.21 (0.18, 149.53) | 0.335 |
|  | **Men** | 0.98 (0.26, 3.75) | 0.975 | 1.43 (0.39, 5.17) | 0.590 | 0.84 (0.35, 2.04) | 0.703 | 0.80 (0.34, 1.88) | 0.610 |
|  | **Women** | 1.22 (0.31, 4.84) | 0.774 | 1.31 (0.41, 4.19) | 0.651 | 1.59 (0.69, 3.64) | 0.272 | 1.10 (0.59, 2.05) | 0.768 |

^a^Risk of NHL associated with log-transformed exposure concentrations stratified by time to diagnosis in cases compared to all controls computed using unconditional logistic regression adjusted for sex, age, centre, phase, batch and sample date

*p<0.05
